# Pyrroline-5-Carboxylate Reductase-2 Promotes Colorectal Cancer Progression via Activating PI3K/AKT/mTOR Pathway

**DOI:** 10.1155/2021/9950663

**Published:** 2021-09-01

**Authors:** Feng Yin, Xiaoxia Huang, Yi Xuan

**Affiliations:** ^1^Department of Laboratory Medicine, Branch of Taian City Central Hospital, Taian, Shandong 271000, China; ^2^Department of Health Care Center, Branch of Taian City Central Hospital, Taian, Shandong 271000, China; ^3^Department of General Surgery Center, Taian City Central Hospital, Taian, Shandong 271000, China

## Abstract

**Aim:**

The aim of this study was to investigate the effect and underlying pathway of pyrroline-5-carboxylate reductase-2 (PYCR2) on colorectal cancer (CRC).

**Methods:**

The Cancer Genome Atlas (TCGA) database was used to analyze PYCR2 expression levels and clinical information. Cell proliferation was evaluated using colony forming and EdU assay. Cell apoptosis rate was determined using flow cytometry. Cell migration and invasion were measured by performing a Transwell assay, and PYCR2, MMP-2, MMP-9, Bax, cleaved caspase-3, Bcl-2, cleaved PARP, p-PI3K, PI3K, p-AKT, AKT, p-mTOR, and mTOR protein levels were detected by Western blot.

**Results:**

A review of the TCGA database revealed that PYCR2 was highly expressed in CRC patients and that high PYCR2 expression was associated with advanced stage, adenocarcinoma, nodal metastasis, and poor survival rate. Moreover, PYCR2 knockdown reduced cell viability, proliferation, migration, and invasion and increased apoptosis. Additionally, PYCR2 knockdown increased Bax, cleaved caspase-3, and cleaved PARP levels and decreased Bcl-2, MMP-2, MMP-9, p-PI3K, p-AKT, and p-mTOR levels in CRC cells. Effects of silencing PYCR2 on proliferation, migration, invasion, apoptosis, and the PI3K/AKT/mTOR pathway in CRC cells were all reversed using a PI3K activator (740Y-P).

**Conclusion:**

PYCR2 was highly expressed in CRC, and its knockdown suppressed CRC tumorigenesis via inhibiting the activation of PI3K/AKT/mTOR pathway. This finding provides a new theoretical foundation for the treatment of CRC.

## 1. Introduction

Colorectal cancer (CRC) is a malignant tumor that occurs in the colon and rectum of the gastrointestinal tract, with an approximate mortality of 600,000 people every year [1]. In fact, CRC has become the world's third largest malignant tumor and the fourth leading cause of cancer-related deaths [2]. This disease develops from adenomatous polyps, undergoing various stages of hyperplasia, adenoma, and carcinogenesis, with corresponding chromosomal changes [3]. However, CRC aetiology remains unclear. In the pursuit of knowledge regarding CRC, the Cancer Genome Atlas (TCGA) database has stored more than 20 cancer genome data types, including mutations, copy number variations, mRNA expressions, miRNA expressions, and methylation data, which are widely used in cancer research [4]. To date, the TCGA has confirmed nine miRNAs and five genes that have prognostic value in colon cancer [5]. Therefore, it is vital to identify novel markers for CRC diagnosis and prognosis and provide new targets for CRC therapy.

Pyrroline-5-carboxylate reductase-2 (PYCR2) is located in the mitochondria and is a vital enzyme in limiting the proline biosynthesis rate [6]. An increasing number of studies have indicated that PYCR2 has an indispensable role in cancer cell proliferation and progression [7]. For example, differential expression of PYCR2 was reported in oesophageal squamous cell carcinoma [8]. As a homologous PYCR2 gene, PYCR1 might also be a new biomarker related to CRC proliferation and drug resistance [9]. However, further investigation is required to elucidate the expression and role of PYCR2 in CRC. In particular, the phosphatidylinosito1-3-kinase (PI3K)/protein kinase B (Akt)/mammalian target of rapamycin (mTOR) signaling pathway is one of the many mechanisms regulating cell cycle and apoptosis, wherein an abnormality in any of this pathway's components may cause tumour development [10]. More specifically, the PI3K/Akt/mTOR signal transduction pathway can induce tumorigenesis via multiple mechanisms: autophagy participation, cell apoptosis inhibition, and tumor suppressor protein p53 downregulation in the nucleus [11, 12]. Given this information, PI3K/mTOR pathway inhibitors have successfully treated primary and metastatic CRC, making this pathway a promising target for clinical cancer treatment [13]. Despite this, however, the effects and underlying pathways of PYCR2 in CRC are still indistinct.

In the current study, we found that PYCR2 knockdown suppressed cell proliferation, migration, and invasion by inhibiting the activation of the PI3K/AKT/mTOR pathway. Thus, we can reasonably predict that PYCR2 may be a potential biomarker of CRC.

## 2. Methods

### 2.1. Cell Culture and Transfection

Human CRC cell lines (LoVo, HT29, HCT8, HCT116, and SW480) and NCM460 cells (Chinese Academy of Sciences Cell Bank, Shanghai, China) were cultured in Dulbecco's modified Eagle's medium (DMEM, Gibco, USA). Meanwhile, siRNA1 and siRNA2 specifically targeting PYCR2 were synthesized and purified by RiboBio (Guangzhou, China), which were then transfected into HCT8 and HCT116 cells using the Lipofectamine 2000 reagent (Invitrogen, Carlsbad, CA, USA). Afterwards, transfection efficiency was determined using quantitative real-time polymerase chain reaction (qRT-PCR). Moreover, HCT8 cells transfected with si2-PYCR2 were cultured in DMEM containing 10 *μ*M PI3K activator (740Y-P, MCE, USA) for 48 h; these cells would be referred to as the si2-PYCR2+740Y-P group.

### 2.2. qRT-PCR

Total RNA was isolated from human CRC cell lines using Trizol (Invitrogen, Carlsbad, CA, USA), and cDNA was synthesized using a cDNA kit (KeyGEN, Shanghai, China). RNA expression levels were then quantified using an ABI qPCR 7500 using a SYBR-Green PCR Master Mix (KeyGEN, Shanghai, China) along with cDNAs and gene-specific primers. The primers used were as follows: PYCR2 forward: 5′-CCTAGCGTTACTTCCGGTCC-3′ and reverse: 5′-AGCCATGAATGCCTTCTCCA-3′; GAPDH forward: 5′-CCACATCGCTCAGACACCAT-3′ and reverse: 5′-AAATGAGCCCCAGCCTTCTC-3′. Additionally, PCR conditions were as follows: 95°C, 10 s (denaturation), 55°C for 30 s (annealing), and 72°C for 30 s (extension) for 40 cycles.

### 2.3. Western Blot

Total proteins were isolated and quantified using a protein concentration detection kit (Beyotime Biotechnology, Shanghai, China). Protein samples were separated using 10% SDS-PAGE and then transferred to polyvinylidene fluoride (PVDF) membranes (Beyotime Biotechnology, Shanghai, China). Nonspecific sites were blocked with 5% milk powder diluted in Tris-buffered saline containing with 0.05% Tween 20 (TBST). Then, the membranes were incubated with the following primary antibodies overnight at 4°C: rabbit polyclonal antibody to PYCR2 (ab103535, 1 : 500, Abcam, UK), rabbit polyclonal antibody to MMP-2 (#40994, 1 : 500, Cell Signaling Technology, USA), rabbit polyclonal antibody to MMP-9 (#13667, 1 : 500, Cell Signaling Technology, USA), rabbit cleaved caspase-3 polyclonal antibody (ab2302, 1 : 500, Abcam, UK), rabbit cleaved PARP monoclonal antibody (#5625, 1 : 500, Cell Signaling Technology, USA), rabbit Bax polyclonal antibody (R-1041-100, 1 : 1000, Biosensis, Thebarton, Australia), rabbit Bcl-2 polyclonal antibody (abs131701, 1 : 500, Absin, Shanghai, China), rabbit p-PI3K polyclonal antibody (#17366, 1 : 200, Cell Signaling Technology, USA), rabbit PI3K polyclonal antibody (#4249, 1 : 500, Cell Signaling Technology, USA), rabbit p-AKT polyclonal antibody (#4060, 1 : 200, Cell Signaling Technology, USA), rabbit AKT polyclonal antibody (pro_37932, 1 : 1000, Bio excellence, Beijing, China), rabbit p-mTOR polyclonal antibody (K008878P, 1 : 200, Solarbio, Beijing, China), and rabbit mTOR polyclonal antibody (abs116084, 1 : 500, Absin, Shanghai, China). For comparison to the control group, rabbit anti-GAPDH polyclonal antibody (1 : 1000, Cell Signaling Technology, USA) was used. After the membranes were washed repeatedly with TBST, they were incubated with horseradish peroxidase- (HRP-) conjugated goat anti-rabbit IgG (#BHR101, 1 : 5000, Beijing Bersee Technology Co. Ltd., Beijing, China). The blots were then assessed using enhanced chemiluminescence, and relative protein levels of PYCR2, MMP-2, MMP-9, Bax, cleaved caspase-3, Bcl-2, p-PI3K, PI3K, p-AKT, AKT, p-mTOR, mTOR, and GAPDH were quantified by densitometry using the Quantity One software (Bio-Rad, Hercules, CA, USA).

### 2.4. PYCR2 Bioinformatics Analysis in CRC

Gene expression data and corresponding clinical information for CRC were downloaded from TCGA, wherein a box diagram was used to show the expression differences of discrete variables. For normalization, the genetic count data in the sample were converted to transcripts per million (TPM) values.

### 2.5. Gene Set Enrichment Analysis

To assess the correlations between PYCR2 expression and tumor-associated pathways, the Gene Set Enrichment Analysis (GSEA) software (version 4.0; http://software.broadinstitute.org/gsea/index.jsp) was used on the TCGA CRC datasets (41 normal samples and 473 CRC samples).

### 2.6. Colony Formation Assay

First, cells were digested into single cells using 0.25% trypsin, which were then suspended for later use. Afterwards, the cells were coated onto plates (1.5 × 10^3^ cells per well) and incubated at 37°C and 5% CO_2_ for 2 weeks. Following incubation, these cells were washed twice with phosphate-buffered saline (PBS), fixed with 5 mL methanol (Sigma, Shanghai, China), and then, stained with 0.1% Giesma solution (Sigma, Shanghai, China) within 10 min. Finally, the dye was washed off with running water, and the cells were air-dried and counted.

### 2.7. EdU Assay

A 5-ethynyl-20-deoxyuridine (EdU) assay kit (Ribobio, Guangzhou, China) was adopted to assess cell proliferation. First, the cells were seeded into confocal plates (10 × 10^5^ cells per well) and then incubated with 50 *μ*M EdU buffer for 2 h at 37°C. Following this, they were fixed with 4% formaldehyde, permeabilized with 0.1% Triton X-100, and then, stained with Hoechst. Finally, the cells were visualized using a fluorescence microscope (Leica, Germany).

### 2.8. Flow Cytometry

First, the transfected cells were suspended and centrifuged for 5 min. Afterwards, these cells were washed and incubated in a buffer solution with FITC-labeled Anexin-V (Carlsbad, CA, USA) for 15 min at 37°C. Next, they were centrifuged for 5 min to remove the supernatant and were then treated with propiridine iodide (PI). Finally, cell apoptosis was evaluated, and cell numbers were calculated using a flow cytometer (FACScan, CA, USA) and CELL Quest 3.0 software (BD Biosciences, Beijing, China).

### 2.9. Transwell Assays

Migration and invasion assays were performed using Transwell chambers following manufacturer's instructions. Briefly, HCT8 and HCT116 cells suspended in serum-free medium were loaded onto the upper chamber, while medium containing 10% FBS was added to the lower chamber. Before migration and invasion assays were performed, the upper chamber was coated with either Matrigel (Millipore, Billerica, USA) for the invasion assay or without Matrigel for the migration assay. After 48 h of incubation at 37°C, the Transwell chamber was removed from the incubator, and the lower surface cells in the upper chamber were stained with crystal violet for 20 min. Finally, the number of migrated and invaded cells was counted under a microscope (Carl Zeiss, Jena, Germany).

### 2.10. Statistical Analysis

Data are presented as mean ± standard deviation (SD) and were analyzed using the SPSS 22.0 (Chicago, IL, USA) and GraphPad Prism 8.0 programs. One-way ANOVA was used for comparisons between groups. Statistical significance was set at *P* < 0.05 for all analyses.

## 3. Results

### 3.1. Correlation between PYCR2 Expression and Clinicopathologic Variables

**Figure 1:**
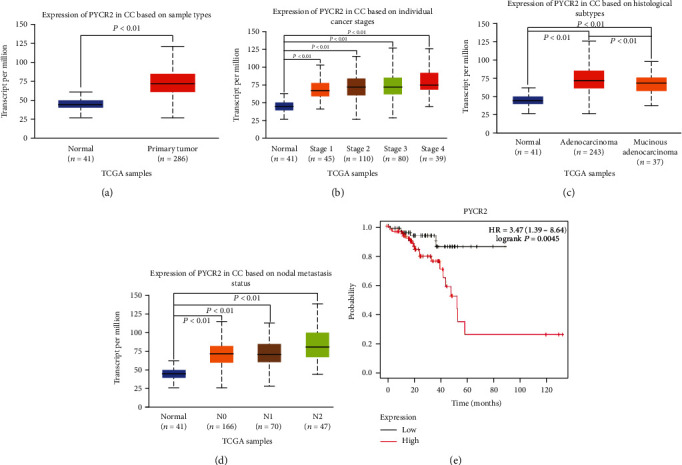
Relation between PYCR2 expression level and clinic characteristics. (a) PYCR2 expression in CRC and normal tissue in TCGA; (b) PYCR2 expression in clinical stage in TCGA; (c) PYCR2 expression in histological subtypes in TCGA; (d) PYCR2 expression in nodal metastasis status in TCGA; (e) PYCR2 expression in overall survival in TCGA.

The correlation between PYCR2 and CRC was analyzed using the TCGA dataset. As indicated in Figure 1(a), PYCR2 expression was significantly higher in primary tumor tissues than in normal tissues (*P* < 0.01, Figure 1(a)), and its expression significantly increased with the increase of cancer staging (*P* < 0.01, Figure 1(b)). Moreover, PYCR2 was highly expressed in adenocarcinoma than in other histological subtypes (*P* < 0.01, Figure 1(c)), and its expression increased significantly with increasing nodal metastases (*P* < 0.01, Figure 1(d)). Furthermore, higher PYCR2 expression was also associated with a lower survival rate (Figure 1(e)). Taken together, PYCR2 was highly expressed in CRC, especially in adenocarcinoma, and higher PYCR2 expression indicated a more advanced stage, increased nodal metastasis, and a poor survival rate compared to that in CRC patients with low PYCR2 expressions.

### 3.2. PYCR2 Was Upregulated in CRC Cells

**Figure 2:**
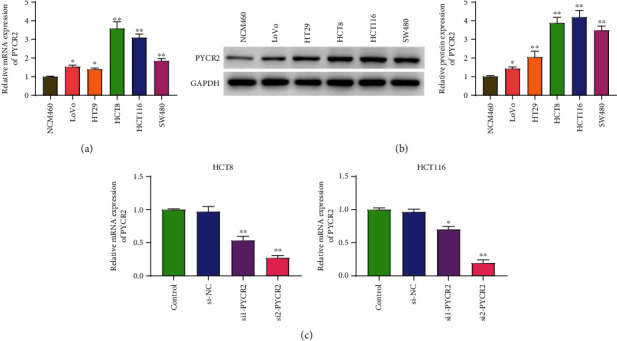
PYCR2 is upregulated in CRC cells. Si-NC group, cells were transfected with si-NC; si1-PYCR2 group cells were transfected with si1-PYCR2; (a) the mRNA level of PYCR2 in was determined by PCR; (b) protein level of PYCR2 was determined by Western blot; (c) transfection efficiency was determined by PCR. ^∗^*P* < 0.05 and ^∗∗^*P* < 0.01 compared with NCM460 cells, ^∗^*P* < 0.05 and ^∗∗^*P* < 0.01 compare with control and si-NC group.

PYCR2 levels in human CRC cell lines (LoVo, HT29, HCT8, HCT116, and SW480) and NCM460 cells were detected by PCR and Western blot. As shown in Figure 2(a), PYCR2 mRNA levels in human CRC cell lines were upregulated as compared to those in NCM460 cells (*P* < 0.01). Simultaneously, Western blot results showed upregulated PYCR2 protein levels in human CRC cells compared to those in NCM460 cells (*P* < 0.01, Figure 2(b)). Collectively, PYCR2 mRNA and protein were upregulated in CRC, especially in HCT8 and HCT116 cell lines. PCR results showed that compared to the control and si-NC groups, PYCR2 mRNA levels were downregulated in si-PYCR2 transfected cells, especially in cells transfected with si2-PYCR2 (*P* < 0.01, Figure 2(c)), indicating a successful transfection process.

### 3.3. PYCR2 Knockdown Attenuated CRC Cell Viability and Proliferation

**Figure 3:**
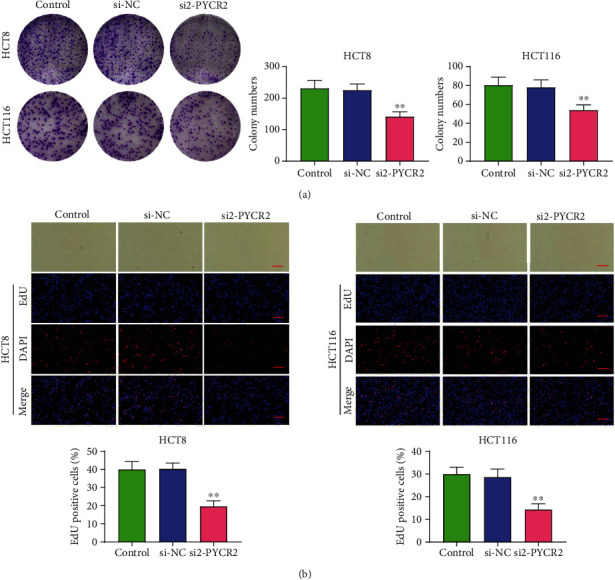
Knock-down of PYCR2 repressed the cell proliferation in CRC cells. Cell proliferation were detected by colony forming assay (a) and EdU assay (b). ^∗∗^*P* < 0.01 compare with control and si-NC group.

The effects of PYCR2 on CRC cell viability and proliferation were evaluated with colony forming assay and EdU assay using HCT8 and HCT116 cells. As indicated in Figure 3(a), cell viability and proliferation were inhibited in cells transfected with si2-PYCR2 (*P* < 0.01). Furthermore, EdU assay results showed that cell viability and proliferation were decreased in cells transfected with si2-PYCR2 compared with those in control and si-NC groups (*P* < 0.01, Figure 3(b)). Taken together, PYCR2 knockdown was found to attenuate CRC cell viability and proliferation.

### 3.4. PYCR2 Knockdown Increased CRC Cell Apoptosis

**Figure 4:**
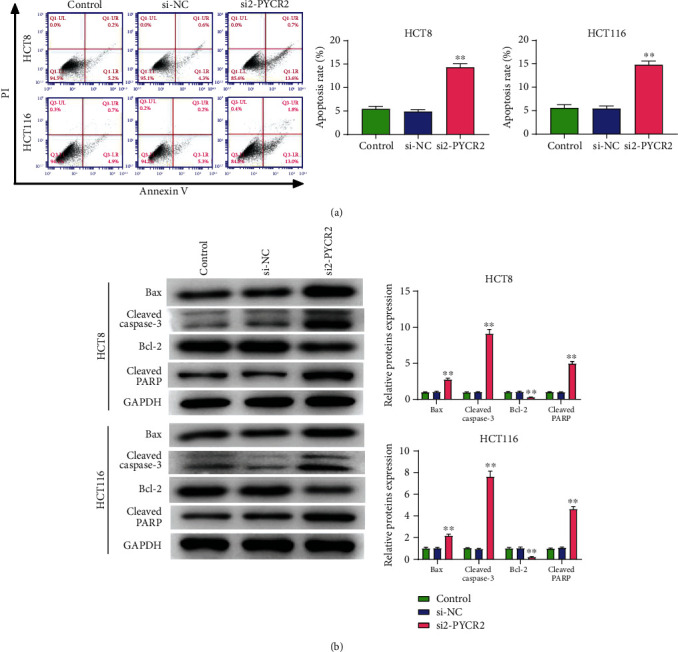
Knock-down of PYCR2 increased CRC cells apoptosis. (a) Cell apoptosis rate was detected by flow cytometry; (b) the apoptosis-related protein (cleaved caspase-3, Bax, Bcl-2, and cleaved PARP) expression level was detected by western blot. ^∗∗^*P* < 0.01 compare with control and si-NC group. ^∗∗^*P* < 0.01 compare with control and si-NC group.

The effects of PYCR2 on apoptosis and apoptosis-related proteins were measured using flow cytometry and Western blot. As shown in Figure 4(a), the apoptosis rate of cells transfected with si2-PYCR2 was significantly higher than that in the control and si-NC groups (*P* < 0.01). Meanwhile, Bax, cleaved caspase-3, and cleaved PARP protein levels were significantly higher, and Bcl-2 protein expression levels were significantly lower in cells transfected with si2-PYCR2 compared to those in the control and si-NC groups (*P* < 0.01, Figure 4(b)). Therefore, the aforementioned results revealed that PYCR2 knockdown increased CRC cell apoptosis.

### 3.5. PYCR2 Knockdown Inhibited CRC Cell Migration and Invasion

**Figure 5:**
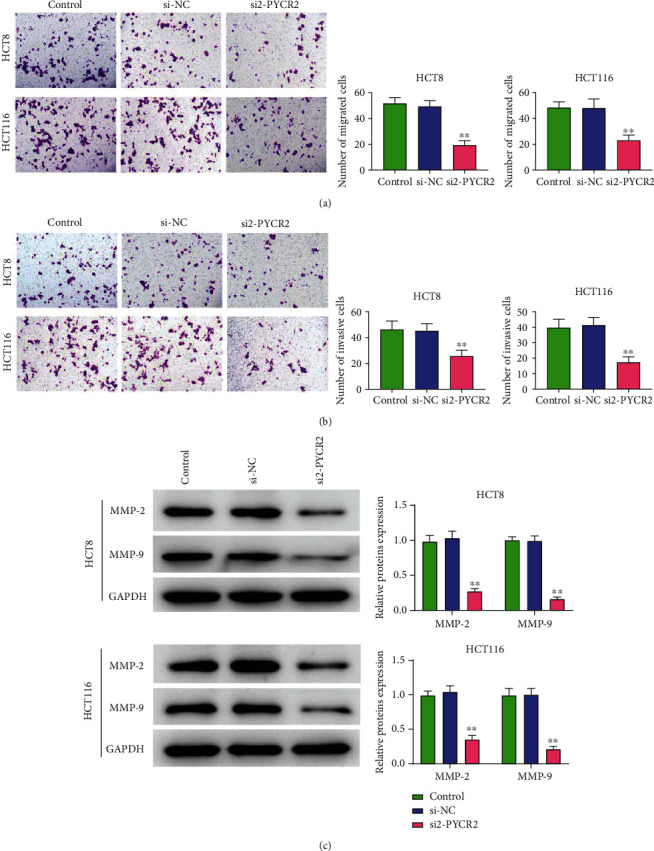
Knock-down of PYCR2 inhibited CRC cells migration and invasion. (a) Cell migration was measured by Transwell assay; (b) cell invasion was measured by Transwell assay; (c) MMP-2 and MMP-9 expressions were detected by Western blot. ^∗∗^*P* < 0.01 compared with control and si-NC group.

The effects of PYCR2 on cell migration and invasion were measured by Transwell assay and Western blot. As indicated in Figure 5(a), the number of migrated cells in the si2-PYCR2 group was lower compared to that in the control and si-NC groups (*P* < 0.01). Moreover, compared with the control and si-NC groups, si2-PYCR2 group showed a significantly lower number of invasive cells (*P* < 0.01, Figure 5(b)). Furthermore, MMP-2 and MMP-9 levels were decreased in the si2-PYCR2 group compared to the control and si-NC groups (*P* < 0.01, Figure 5(c)). Thus, PYCR2 knockdown inhibited CRC cell migration and invasion.

### 3.6. PYCR2 Knockdown Inhibited the Activation of PI3K/AKT/mTOR Pathway in CRC Cells

**Figure 6:**
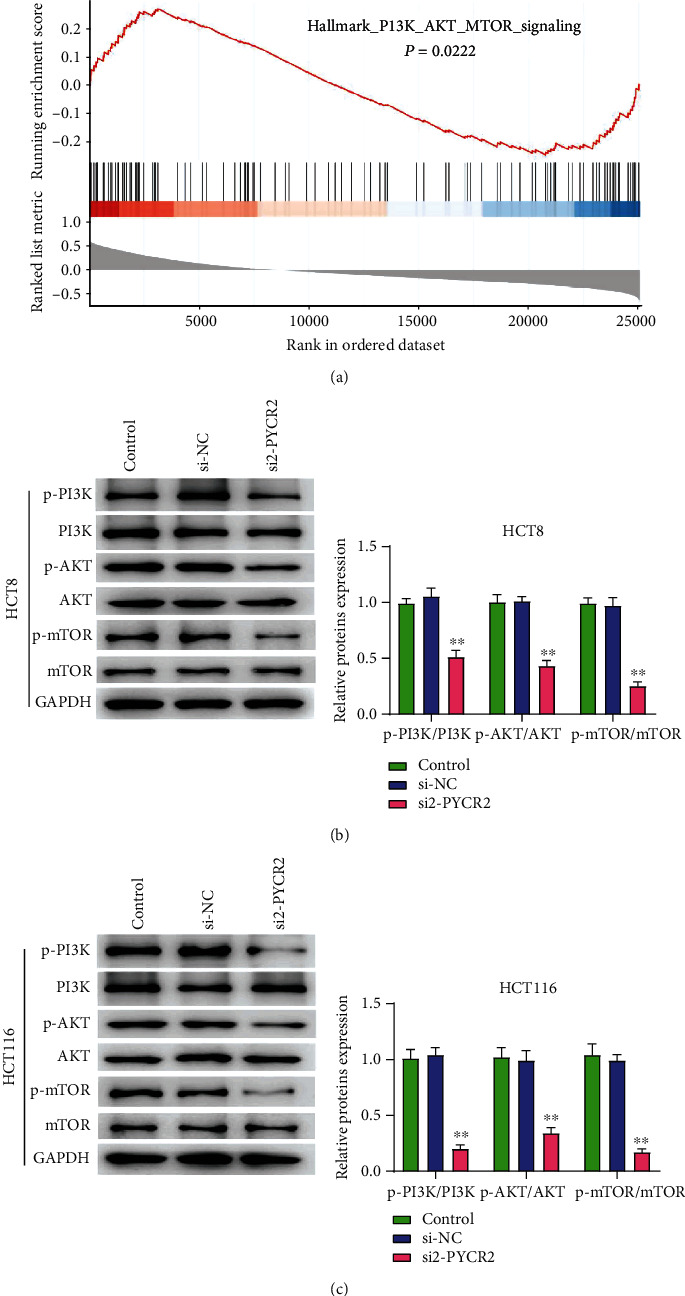
PYCR2 regulated CRC via PI3K/AKT/mTOR pathway. (a) Enrichment plots from gene set enrichment analysis; (b) the levels of p-PI3K, PI3K, p-AKT, AKT, p-mTOR, and mTOR in HCT8 cells were detected by Western blot; (c) the levels of p-PI3K, PI3K, p-AKT, AKT, p-mTOR, and mTOR in HCT116 cells were detected by Western blot. ^∗∗^*P* < 0.01 compared with control and si-NC group.

**Figure 7:**
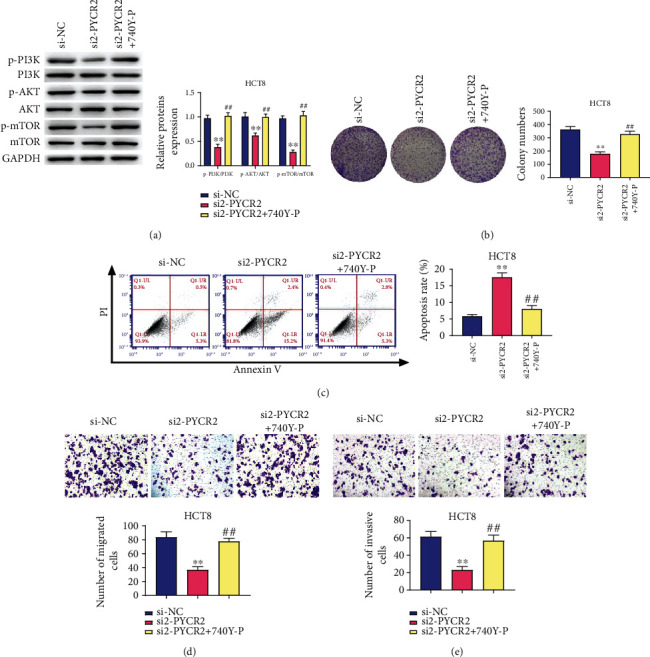
740Y-P reversed the functions of silencing PYCR2 on the proliferation, migration, invasion, and apoptosis of HCT8 cells. (a) The levels of p-PI3K, PI3K, p-AKT, AKT, p-mTOR, and mTOR in HCT8 cells were detected by western blot; (b) cell proliferation was detected by colony forming assay; (c) cell apoptosis rate was detected by flow cytometry; (d) HCT8 cell migration was measured by Transwell assay; (e) HCT8 cell invasion was measured by Transwell assay. ^∗∗^*P* < 0.01 compare with si-NC group; ^##^*P* < 0.01 compared with si2-PYCR2 group.

GSEA was used to assess the correlations between PYCR2 expression and tumor-associated pathways in CRC through analyzing the TCGA CRC datasets (41 normal samples and 473 CRC sample). As shown in Figure 6(a), the PI3K/AKT/mTOR pathway was enriched with high PYCR2 expression phenotype (*P* < 0.05), indicating that the PI3K/AKT/mTOR pathway could be involved in the cancer-promoting effect of activated PYCR2. In particular, p-PI3K/PI3K, p-AKT/AKT, and p-mTOR/mTOR expressions were downregulated after silencing PYCR2 (*P* < 0.01, Figures 6(b) and 6(c)), whereas 740Y-P treatment significantly reversed this downregulation in HCT8 cells (*P* < 0.01, Figure 7(a)). Additionally, 740Y-P treatment significantly reversed the inhibitory function of silencing PYCR2 on HCT8 cell proliferation (*P* < 0.05, Figure 7(b)), migration (*P* < 0.01, Figure 7(d)), and invasion abilities (*P* < 0.01, Figure 7(e)). Flow cytometry, on the other hand, showed that 740Y-P treatment reversed the effects of silencing PYCR2 on HCT8 apoptotic cells (*P* < 0.01, Figure 7(c)). These results indicated that PYCR2 knockdown inhibited the activation of PI3K/AKT/mTOR pathway in CRC cells.

## 4. Discussion

Owing to the lack of a standardized CRC screening program, most CRC patients present to the doctor when already in the metastatic stage, with a high recurrence rate even after surgery [14]. Therefore, the prognosis of CRC patients cannot be effectively improved only on the basis of conventional treatment methods, without the imperative molecule-targeting therapies [15]. Currently, the most important steps for the development of such therapies are the discovery of molecular biomarkers for disease prognosis and determination of the mechanisms underlying CRC formation and progression. To date and to our knowledge, we are the first to investigate the role of PYCR2 in CRC. More specifically, it was found that PYCR2 was highly expressed in CRC based on analysis of the TCGA data. Increase in cancer staging and metastasis was also associated with an increase in PYCR2 expression levels. In addition, increased PYCR2 expression was associated with shortened overall survival time.

A special effect of proline in metabolic regulation is now accepted, and recent studies have confirmed that proline metabolism is critical in cancer reprogramming [16]. Because proline metabolism is catalysed by PYCR2, it was speculated that PYCR2 was a potential molecular target for cancer treatment [17]. First, PYCR2 expression level was investigated in all cell lines, with results showing that PYCR2 was upregulated in CRC cells, which is consistent with TCGA data analysis. Cancer is the result of malignant proliferation of cells [18]. in our study PYCR2 knockdown inhibited cell proliferation of CRC cells, indicating that PYCR2 plays an important role in CRC.

To further understand the potential molecular mechanism of PYCR2 in CRC development, the effects of PYCR2 on cell apoptosis, migration, and invasion were investigated. Apoptosis is an active process, involving the activation and regulation of a series of proteins, such as caspase-3, Bax, and Bcl-2 [19, 20], and is a vital mechanism for the occurrence and development of CRC. PYCR2 silencing, in particular, was found to induce cell apoptosis, which was mainly manifested by increased levels of proapoptotic factors (cleaved caspase-3, Bax, and cleaved PARP) and reduced levels of antiapoptotic factors (Bcl-2). Studies have shown that matrix metalloproteinases (MMPs) mediate cancer cell migration and invasion by extracellular matrix (ECM) degradation [21, 22]. We found that PYCR2 knockdown inhibited cell migration and invasion, as well as MMP-2 and MMP-9 expressions. Similarly, Ou et al. found that silencing PYCR2 inhibited cell apoptosis, migration, and invasion of melanoma cells [17]. Based on the findings from the present and previous studies, PYCR2 could be said to be involved in CRC development, but its underlying mechanism needs to be studied further.

A growing body of evidence has indicated that the PI3K/AKT/mTOR pathway participates in modulating cellular events, such as cell growth, adhesion, migration, and survival [23–25]. Some studies have confirmed that inhibition of the PI3K/Akt/mTOR pathway is of great significance for CRC treatment [26, 27]. The PI3K/Akt pathway can inhibit CRC cell apoptosis through its effects on caspase, Bcl, FOXO, and mTOR genes [23, 28]. mTOR activation, in particular, mediates downstream molecules of the PI3K/Akt/mTOR signaling pathway, subsequently affecting cell growth, apoptosis, and metabolism [29, 30]. Moreover, Ou et al. [17] have suggested that silencing PYCR2 can decrease the expression level of mTOR. Intriguingly, the results of GSEA of TCGA datasets showed that the cancer-promoting effect of PYCR2 was closely associated with the PI3K/AKT/mTOR pathway in CRC. Thus, we explored the association between PYCR2 and the PI3K/AKT/mTOR pathway in subsequent experiments. Our results confirmed that silencing PYCR2 inhibited the activation of the PI3K/Akt/mTOR pathway in CRC cells. Moreover, 740Y-P was found to reverse the functions of silencing PYCR2 on CRC cell proliferation, migration, invasion, and apoptosis.

## 5. Conclusions

In conclusion, PYCR2 knockdown suppressed cell proliferation, migration, and invasion via inhibiting the activation of the PI3K/AKT/mTOR pathway in CRC cells, thus providing a potential novel therapeutic target for the diagnosis and treatment of CRC.

## Data Availability

The datasets used and analyzed during the current study are available from the corresponding author on reasonable request.
